# Viscount Knutsford's Letter

**Published:** 1918-10-12

**Authors:** 


**[Correspondence on all subjects is invited, but we cannot in any way be responsible for the opinions expressed by our correspondents, who must give their name and address as a guarantee of good faith, but not necessarily for publication. Correspondents are reminded that brevity of style and conciseness of statement greatly facilitate early insertion.]**

### Viscount Knutsford's Letter.

*To the Editor of* The Hospital.

Sir,—I hoped to have returned from my holiday clothed with new vigour and strength. I shall return clothed in sackcloth and ashes. I learn that the treatment of my nurses is "monstrous, callous and cruel." Forgive the word "my"; I have cared for them so much. So be it. It must be so because Major Chapple, of New Zealand, says so, and Major Chapple is an "honourable" man. So are they all (all M.P.s) "honourable" men. But they sometimes are inaccurate and write about what they know nothing.—Yours truly,

Knutsford.

